# Biochemical Characterization of *Paracoccidioides brasiliensis α*-1,3-Glucanase Agn1p, and Its Functionality by Heterologous Expression in *Schizosaccharomyces pombe*

**DOI:** 10.1371/journal.pone.0066853

**Published:** 2013-06-25

**Authors:** Héctor Villalobos-Duno, Gioconda San-Blas, Maryan Paulinkevicius, Yolanda Sánchez-Martín, Gustavo Nino-Vega

**Affiliations:** 1 Centro de Microbiología y Biología Celular, Instituto Venezolano de Investigaciones Científicas, Caracas, Venezuela; 2 Instituto de Biología funcional y Genómica and Departamento de Microbiología y Genética, Universidad de Salamanca, Salamanca, Spain; University of Cambridge, United Kingdom

## Abstract

α-1,3-Glucan is present as the outermost layer of the cell wall in the pathogenic yeastlike (Y) form of *Paracoccidioides brasiliensis*. Based on experimental evidence, this polysaccharide has been proposed as a fungal virulence factor. To degrade α-1,3-glucan and allow remodeling of the cell wall, α-1,3-glucanase is required. Therefore, the study of this enzyme, its encoding gene, and regulatory mechanisms, might be of interest to understand the morphogenesis and virulence process in this fungus. A single gene, orthologous to other fungal α-1,3-glucanase genes, was identified in the *Paracoccidioides* genome, and labeled *AGN1*. Transcriptional levels of *AGN1* and *AGS1* (α-1,3-glucan synthase-encoding gene) increased sharply when the pathogenic Y phase was cultured in the presence of 5% horse serum, a reported booster for cell wall α-1,3-glucan synthesis in this fungus. To study the biochemical properties of *P. brasiliensis* Agn1p, the enzyme was heterologously overexpressed, purified, and its activity profile determined by means of the degradation of carboxymethyl α-1,3-glucan (SCMG, chemically modified from *P. brasiliensis* α-1,3-glucan), used as a soluble substrate for the enzymatic reaction. Inhibition assays, thin layer chromatography and enzymatic reactions with alternative substrates (dextran, starch, chitin, laminarin and cellulose), showed that Agn1p displays an endolytic cut pattern and high specificity for SCMG. Complementation of a *Schizosaccharomyces pombe agn1*Δ strain with the *P. brasiliensis AGN1* gene restored the wild type phenotype, indicating functionality of the gene, suggesting a possible role of Agn1p in the remodeling of *P. brasiliensis* Y phase cell wall. Based on amino acid sequence, *P. brasiliensis* Agn1p, groups within the family 71 of fungal glycoside hydrolases (GH-71), showing similar biochemical characteristics to other members of this family. Also based on amino acid sequence alignments, we propose a subdivision of fungal GH-71 into at least five groups, for which specific conserved sequences can be identified.

## Introduction

Paracoccidioidomycosis (PCM) is a human systemic mycosis caused by four species, comprised by the *Paracoccidioides brasiliensis* complex (S1, PS2 and PS3; [1]) and *Paracoccidioides lutzii*, a recently described species, so far reported only in Brasil [2]. Confined geographically to Latin America, where it is one of the most frequent systemic mycoses, PCM may result in a fatal outcome [3]. In *P. brasiliensis*, changes in cell wall composition associated to the thermal dimorphism exhibited by this fungus, are closely related to pathogenicity and virulence [4]. Experimental evidence suggests that *P. brasiliensis* cell wall α-glucan, the fungal outermost layer, plays a protective role against host defense mechanisms [5]. Later studies in *Histoplasma capsulatum* [6], confirmed San-Blas and San-Blas’ findings [5] with regard to the importance of α-1,3-glucan as a virulence factor. Furthermore, the wide layer of α-1,3-glucan in *H. capsulatum* yeast cell wall hides the underlying β-1,3-glucan, preventing in this way its efficient exposure to macrophages, and impairing the secretion of TNFα. As a result, the immune response of the infected organism is reduced [7]. The absence of α-1,3-glucan in mammalian cells, raises the possibility of developing specific antifungal drugs targeted toward the blockage of α-1,3-glucan biosynthesis, which might result in depression of fungal virulence, allowing the natural immune response of the infected organism towards the fungus, and preventing the disease.

α-1,3-Glucan has been found in a few fungal species such as *Schizosaccharomyces pombe*, *P. brasiliensis*, *H. capsulatum*, *Blastomyces dermatitidis*, *Cryptococcus neoformans* and *Aspergillus* species [6], [8], [9], [10], [11]. Experimental data in *S. pombe* demonstrate that during vegetative growth, the cell wall α-1,3-glucan is built with two linear glucose polymers, each 260 residues-long, interconnected through α-1,3 and α-1,4 glycosidic linkages [9]. In *P. brasiliensis*, α-1,3-glucan is composed of a single linear polymer of α-1,3 linked- glucose residues, and occasional ramifications of one glucose moiety bound to the backbone by α-1,4 linkages [12].

α-1,3-Glucanases (EC 3.2.1.59), also called mutanases due to their ability to degrade the extracellular glucan synthesized by the bacterium *Streptococcus mutans* [13], are enzymes capable of hydrolyzing glucose polymers linked by α-1,3 glycosidic bonds. According to their amino acid sequence, these enzymes are grouped into the family 71 of glycoside-hydrolases (GH-71). Depending on the final products, either oligo- or monosaccharides, they are divided into endolytic or exolytic enzymes [14].

In *S. pombe*, two α-1,3-glucanase genes are present (*agn1* and *agn2*), whose translation products Agn1p and Agn2p are involved in different cell processes. Agn1p is involved in cytokinesis [15]. *S. pombe agn1* mutants are unable to separate as free cells, impairing the physical division of the cell during cell fission [15], [16]. Meanwhile, Agn2p is involved in the process of sexual differentiation, sporogenesis or spore formation, specifically in the process of ascospore release, as demonstrated by its inhibition in *S. pombe agn2* mutants [17].

After the exhaustion of glucose, *A. nidulans* α-1,3-glucanase is secreted to the cell wall and mobilizes α-1,3-glucan, the main reserve material accumulated during vegetative growth in the cell wall; once monosaccharides are released, they are captured and metabolized by the cell during starvation [18]. In *Trichoderma harzianum,* α-1,3-glucanase degrades cell wall of plant pathogenic fungi, thus becoming an inhibitor of spore germination and mycelial growth of a wide range of fungal pathogens [19]. Additionally, in fungi the morphological changes associated with extensive alterations in cell wall composition are regulated by the action of polysaccharide synthases and hydrolases. These enzymes may facilitate the complex patterns of lysis, branching and cross-linking of glucans involved in the process of fungal wall synthesis.

As a further step into the comprehension of the cell wall α-1,3-glucan metabolism in *P. brasiliensis*, we aimed to characterize the *P. brasiliensis* α-1,3-glucanase by heterologous expression of its encoding gene, *AGN1*, and purification of its transcriptional product, Agn1p. Functionality of the gene was assessed by complementation of an *S. pombe agn1*Δ mutant with the *P. brasiliensis AGN1* gene.

## Materials and Methods

### Strains and Growth Conditions

*P. brasiliensis* Pb73 (ATCC 32071), was grown on liquid YPD (0.5% w/v bactopeptone, 0.5% w/v yeast extract, 1.5% w/v glucose), either at 23°C (M cultures) or 37°C (Y cultures) with or without 5% horse serum (Gibco) with continuous shaking at 100 rpm for 3–4 days. *Escherichia coli* QIAGEN EZ chemically competent cells (Qiagen, Hilden, Germany), used for propagation of plasmids and cloning experiments was grown in Luria–Bertani (LB) medium (0.5% w/v yeast extract, 1% w/v triptone, 1% w/v NaCl) and supplemented with 100 µg/ml ampicillin (Sigma-Aldrich, St Louis, MO, EE.UU) when required for plasmid selection. *E.coli* M15 [pREP4] (Qiagen, Hilden, Germany), used for heterologous expression and Agn1p purification, was grown in LB medium with 25 µg/ml kanamycin (Sigma-Aldrich, St Louis, MO, EE.UU) and supplemented with 100 µg/ml ampicillin (Sigma-Aldrich, St Louis, MO, EE.UU) for plasmid selection. *Schizosaccharomyces pombe*, strains wt-64 (*leu* 1–32, *his3*D1, *ura*D18, *ade6*m210h^−^) and 1252 (*agn1*::*ura4*+, *leu* 1–32, *his3*D1, *ura*D18, *ade6*m210h^−^) [16], were grown for maintenance and storage in YES medium [20]. For complementation experiments of strain 1252 (*S. pombe agn1* mutant), Edinburgh minimal medium (EMM) was employed [20]. Cells were grown at 30°C and 120 rpm, between 24 and 48h. All cells were observed before use in a phase contrast microscope to discard contamination (Nikon Optiphot, Japan).

### Nucleic Acids Isolation

Genomic DNA (gDNA) extraction was performed as previously described [21]. RNA was obtained from freeze-dried macerated cells of *P. brasiliensis* using TRIzol Reagent™ (GIBCO Life Technologies, Rockville, USA), following the manufacturer's instructions. *S. pombe* gDNA from wild type strain wt-64, or plasmid DNA from *S. pombe* strain 1252, were isolated according to Hoffman and Winston [22]. The AxyPrep Multisource Total Miniprep Kit (Axygen Biosciences) was used for extraction of total RNA from the mutant strain 1252, following the manufacturer's recommendations.

### Isolation and Sequencing of AGN1

For isolation of *P. brasiliensis AGN1*, a *Hin*dIII partial genomic library was constructed as follows: 100 µg of *P. brasiliensis* DNA were digested with *Hin*dIII (Invitrogen), and size-fractionated fragments (according to Southern analysis) were cloned into pBluescript SK vector (Stratagene). Resulting transformants were collected and screened by colony hybridization, with a 750 bp PCR amplified fragment of the putative *H. capsulatum AGN1* gene, by using Mut(F): 5′ ATY GAY GCA TTY GCW CTY AAY 3′ and Mut (R): 5′ GAY TCR CCG TAG TC 3′, primers. A positive clone yielding plasmid pMP1, containing a 2.3 kb insert was isolated and sequenced, showing to contain a partial sequence of the gene. The complete *P. brasiliensis AGN1* gene sequence was obtained using the SMART™ RACE cDNA amplification kit (Clontech Laboratories, Inc., Mountain View, CA, USA). The following primers were used as gene-specific primers: HV1 (5′-GTA CCA GAA TGT GAT AAT GTC GGC GG-3′) and HV2 (5′-GCT GGA CAA ATT CTG GCT GTA GTG TG-3′) towards the 5′ region and GlnF (5′ AGT TTT GGG TCA TAA GCC G 3′) towards the 3′ region. cDNA was amplified by RT-PCR, and the amplicons cloned into vector pDrive (QIAGEN GmbH, Hilden, Germany) for sequencing. For sequencing reactions, plasmid preparations were carried out with the Concert™ Miniprep System (Life Technologies, Carlsbad, CA, USA) or Axyprep Plasmid Miniprep Kit (Axygen Biosciences, Union City, CA, USA). Nucleotide sequencing was automated on an ABI PRISM 377 DNA sequencer (Perkin Elmer) (Unidad de Estudios Genéticos y Forenses (UEGF), Centro de Microbiología y Biología Celular, IVIC, Caracas, Venezuela). The sequence has been deposited in GenBank (accession number EF679780).

### Computer-assisted Sequence Analyses

Assembly of the nucleotide sequences and translated protein sequences were generated with the Vector NTI suit package (Vector NTI, InforMax, Inc., USA). Homology searches were performed on the GenBank database using BLAST 2.0 [23]. Domain analyses of Agn1p were performed using SMART internet service for sequence analyses and prediction of protein structure and function [24], identification of protein patterns and profiles with PROSITE [25], and FASTA for proteins, at the The European Bioinformatics Institute-web site (EMBL-EBI) [26]. Molecular weight and isoelectric point were calculated with the Compute pI/Mw tool [27]. SignalP 3.0 (Center for Biological Sequence Analysis, CBS; [28]) was used for signal peptide prediction. The protein hydrophobicity plots were done according to Kyte and Doolitle [29], using the program hosted at the web site http://www.vivo.colostate.edu/molkit/hydropathy/. The genome database of *P. brasiliensis* (http://www.broadinstitute.org/) was used to verify the presence of one or more genes belonging to the family 71 of glycoside hydrolases. The MEGA 4 software was used for sequence alignment (using ClustalW) and the construction of phylogenetic tree was done by the neighbor-joining method.

### Quantitative RT-PCR

Total RNA was treated with DNase by using the TURBO DNA free™ kit (Ambion Inc., Austin, TX, USA). The RETROScript™ kit (Ambion Inc., Austin, TX, USA) was used for reverse transcription of mRNA. For real-time PCR of *AGN1*, primers RT3∶5′-GCA GCA AGT TAT CAC ACT AC-3′ and RT4∶5′-TGG TTC CGT CAT ACA TTT TA-3′ were used. For expression analysis of *AGS1*, sequence specific primers FrwAGS1_RT: 5′-AAA TGC GGC ACG GAG GAG A-3′ and RevAGS1_RT: 5′-AAG GGT GGT ATC AAG TGC CGA GT-3′ were used. To find the best internal control as normalizer for the expression experiments, two genes were used. Amplification of *18S* rRNA was carried out, using the primers 18S S3∶5′-CGA TTC CGG AGA GGG AGC C- 3′ and 18S AS3∶5′-CGT ATC GGG ATT GGG TAA TTT GC-3′. A second reference gene (*Pbl34*) which has no changes in transcription on both morphologies [30] was also analyzed, using the primers designed by Moreira-Dantas [30]. In experiments aimed to evaluate the changes induced by horse serum, changes in expression levels of the *18S* gene were observed. Therefore, the *Pbl34* gene was chosen as the normalizer gene for all subsequent experiments. Quantitative PCR was performed in triplicate on an iQ5 real time PCR detection system, using the GoTaq® qPCR Master Mix (Promega Corporation, Madison, WI, EE.UU), in a 15 µl volume (7.5 µl Master Mix 2X, 5.5 µl of a forward and reverse primer mix 0.2 µM, and 2 µl cDNA). Reaction conditions were as follows: 95°C for 3 min, followed by 40 cycles at 94°C for 10 s, 58°C for 30 s, and 72°C for 30 s, with dissociation conditions of 95°C for 1 min, 55°C for 1 min, and 81 cycles starting at 55°C, with temperature increases of 0.5°C every 10 s up to 95°C. PCRs with serial dilutions of *P. brasiliensis* cDNA as template were used to calculate the amplification efficiency for each pair of primers. All Ct values were normalized to the Ct values of the standard gene and the relative expression levels were calculated using the 2^−ΔΔCT^ method [31]. Statistical analysis of the data was done by comparing their mean expression levels, using the Turkey-Kramer test, included in the InStat statistical package (GraphPad Software).

### Heterologous Expression and Purification of *P. brasiliensis* Agn1p

For Agn1p-his purification, the *P. brasiliensis AGN1* ORF (Gen-Bank Accession No. EF679780) deprived of the sequence coding for the putative signal peptide was PCR-amplified from cDNA, using primers HVC2 5′-CAT AGA GCT CAT TCA AAC ATC CAC GCT-3′ and HVC3 (5′-GGA TCC AAG GCT GTA TTT GCC CAT TTC-3′), and cloned at the *Sac*I and *Bam*HI restriction sites of plasmid pQE30Xa (Qiagen), generating pHV2. *E.coli* M15 [pREP4] (Qiagen, Hilden, Germany) was transformed with pHV2 or pQE30Xa empty (as negative control), grown on LB medium supplemented with 100 µg/ml ampicillin (Sigma-Aldrich, St Louis, MO, EE.UU) and 25 µg/ml kanamycin (Sigma-Aldrich, St Louis, MO, EE.UU) at 37°C, following the manufacturer’s indications. For protein expression experiments, each transformant was grown on LB medium supplemented with 100 µg/ml ampicillin (Sigma-Aldrich, St Louis, MO, EE.UU), 25 µg/ml kanamycin (Sigma-Aldrich, St Louis, MO, EE.UU), 500 mM NaCl, 0.2% glucose and 1 mM sorbitol at 37°C for 5h until culture OD_600_ reached 0.7. Protein expression was induced with the addition of 0.5 mM IPTG and cultures were grown at 23°C overnight [15], [32]. Cells were harvested by centrifugation (20 min, 4000g, 4°C) and washed with lysis buffer (50 mM NaH_2_PO_4_, 300 mM NaCl, 10 mM imidazole, pH 8.0). Cell pellets were resuspended in lysis buffer supplemented with 1 µl/ml protease inhibitor cocktail (Sigma P-8215), treated with 1 µg/ml lysozyme (Sigma L-6876) on ice for 30 min. Cells were lysed with 0.17 µm glass beads [33], in a Braun homogenizer (Braun, Melsungen, Germany), using 4 pulses of 1 min each, with 1 min cooling on ice between pulses. Cell debris was removed by centrifugation at 4°C at 10000×g for 15 min. Clear lysates were incubated with pre-washed nickel-NTA resin (QIAGEN) at 4°C for 1 h, and subsequently washed with buffer (50 mM NaH_2_PO_4_, 300 mM NaCl, 20 mM imidazole, pH 8.0). Agn1p-his was eluted in fractions by addition of elution buffer (50 mM NaH_2_PO_4_, 300 mM NaCl, 250 mM imidazole, pH 8.0). The eluates were pooled and concentrated to 1 ml using Amicon ® Ultra-4 (Millipore) with a 30 kDa cutoff. The concentration process was performed at 4000g for 90 min at 4°C. Purity was monitored by SDS-PAGE analysis employing Mini-PROTEAN chambers ® II Electrophoresis Cell (Bio-Rad, Hercules, CA, USA), as recommended by the manufacturer and according to the size of the expected product [34], [35]. Separation and stacking gels of 10 or 4% polyacrylamide were used. The following molecular weight standards were employed: Prestained marker (98,5 - 14) kDa (26041-020, Gibco-BRL) and 6xHis Protein Ladder (100-15) kDa (34705, QIAGEN). Immunodetection of the purified protein was performed with the chromogenic method described in the QIAexpress ® Detection System manual (Qiagen, Hilden, Germany), using the HRP Conjugate Kit RGS-His.

### Extraction and Solubilization of α-1,3-glucan

In order to test the enzymatic activity and specificity of *P.* *brasiliensis* Agn1p, *P. brasiliensis* yeast-like cell wall was extracted by alkaline separation as in [36]. The alkali-insoluble α-1,3-glucan was converted into soluble carboxymethyl-α-1,3-glucan (SCMG) to ensure its availability in aqueous solution for enzymatic assays. For this, monochloroacetic acid was used in basic medium to modify the hydroxyl group of carbon 6 [37], [38]. Briefly, 318 mg of α-1,3-glucan were resuspended in 10 ml 2-propanol with stirring for 30 min at room temperature. Next, 0.5 ml of 30%NaOH was added dropwise for 60 min with agitation. The mix was vigorously stirred for 90 min, after which 381 mg monochloroacetic acid were added. The reaction was stirred for further 4 h at 65°C in a Heidolph MR 3001 K thermocouple (© Heidolph Instruments GmbH & Co. KG). The product was recovered by filtration on Whatman # 1, and washed successively with methanol-acetic acid (7∶3 v/v), methanol-water (4∶1 v/v), methanol and a final step with acetone. The supernatant was filtered again and washed with acetone, allowed to dry overnight, followed by suspension in 150 ml of distilled water, dialyzed overnight against water and finally lyophilized to complete dryness.

### Infrared (IR) Spectroscopy

Samples were prepared as KBr pellets. IR spectra were recorded from 3500 to 500 cm^−1^, using a Nicolet iS10 IR spectrometer (Thermo Fisher Scientific, Waltham, MA, EE.UU), coupled to the OMNIC 8.0 software.

### NMR Analysis

^13^C-NMR experiments were carried out either in a Bruker 300 or Bruker 500 Ultrashield spectrometers at 75 MHz and 125 MHz. The polysaccharides (20 mg) were dissolved in D_2_O (1 ml) using a data collection time of 16 h [39], according to the indication of the Nuclear Magnetic Resonance Service, Center of Chemistry, IVIC.

### Enzymatic Assays

All reactions were carried out with 100 µg of Agn1p-his and 1 mg/ml of SCMG in CH_3_COONa buffer (50 mM, 1 h) in a final volume of 1 ml. Reactions were stopped by heating at 100°C [40]. Free reducing ends were analyzed using the colorimetric bicinchoninic acid (BCA) assay [41]. Optimum pH and optimum temperature were determined by performing the reaction at pH values between 4.0 and 7.2, and a temperature range between 23 to 50°C, respectively.

To test the effect of inhibitors, 1-deoxynojirimycin (Sigma, D9305), D-glucono-1,5-lactone (Sigma, G-9766), or 50 mM CH_3_COONa buffer pH 5.0 were preincubated on ice for 15 min with Agn1p-his or glucoamylase (0.5 µg/ml) (Roche 1202332), as a positive control of inhibition. Remaining Agn1p-his activity was measured by incubation with SCMG (1 mg/ml) in optimal conditions (final inhibitor concentration, 250 µM). Remaining glucoamylase activity was measured in 50 mM sodium acetate, pH 5.6, containing 1 mg/ml of starch (Sigma, S2004) [42]. Hydrolysis products were analyzed using the colorimetric BCA assay [41].

Substrate specificity was determined by incubation at optimal conditions of Agn1p-his with substrates at a final concentration of 1 mg/ml. Carboxymethylated α-(1,3)-glucan (SCMG), starch (Sigma, S2004), carboxymethyl-chitin (Carbomer, 5-00934), carboxymethyl-laminarin (Carbomer, 5-02294), carboxymethyl-cellulose (Sigma, C-8758), and dextran T500 (Pharmacia), were used as substrates. Reaction products were analyzed colorimetrically [15], [41]. One unit of glucanase activity was defined as the amount of enzyme needed to generate one µmol of reducing ends per minute at optimal pH and temperature.

The enzymatic reactions were concentrated to 50 µl by lyophilization. Aliquots of 5 µl were placed on a TLC plate (EMD, 5715-7, TLC Silica Gel 60 F254 20x20 cm), using propanol/water (7/3, v/v) as the mobile phase, for three hours in a closed chamber previously saturated with the solvent mixture. As standard markers, glucose (Himedia, RM 016-500G), maltose (Sigma, M5885), maltotriose (Sigma, 851,493) at a concentration of 333 µg/ml were placed, in separate contiguous lanes. After completion of the run, the plates were dried at 60°C for 10 min, and impregnated with iodine vapors or a specific staining solution for carbohydrates (KMnO_4_ 1.5 g; K_2_CO_3_ 10 g, 1.25 ml NaOH 10% in 200 ml of distilled water) using the spray-type sprayer Flask Aldrich (Z190373 -1EA). Plates were left to dry and developed for about 1 h at 60°C, producing yellow spots on a pink background [43]. The Rf for each lane was calculated by the ratio of the distances traveled by the spots to the distance reached by the front.

### *S. Pombe* Complementation Assay

Two constructions were prepared for use in complementation tests: The first one was obtained by cloning the *P. brasiliensis AGN1* ORF into the *Xho*I and *Bam*HI restriction sites of the *S. pombe* expression vector pREP3X [44], [45], generating plasmid pHV3. The second one, was achieved by replacing the *P. brasiliensis AGN1* signal peptide coding sequence from its ORF, with the *S. pombe agn1* signal peptide coding sequence, by means of PCR overlap extension [46], using Advantage ® 2 DNA Polymerase Mix (Clontech Laboratories, Inc.) and cloning the resulting chimera into the *Xho*I and *Bam*HI restriction sites of expression vector pREP3X, to produce plasmid pHV4. Oligonucleotide sequences used for amplification of both products can be found in Table 1.

**Table 1:** Oligonucleotides used in the amplification of PCR products for the complementation of *S. pombe.*

Name	Sequence	Target
AgnCOMPFw (*XhoI*)	5′-CTCGAGATGCGTCTAAAATATCTCTTTTCA-3′	*AGN1* 1
AgnCOMPRv (*BamHI*)	5′-GGATCCTCAAACATCCACGCTGGACCCAAC-3′	
a: PsPombeFw	5′-CTCGAGATGAAGCTTGTGCTATTTCTG-3	Ps*agn1::AGN*T^2^
b: PSp-AgnpbTRv	5′-TGGGCAAATACAGCCTTAGCGTTAGTCAAATT-3′	
c: PSp-AgnpbTFw.	5′-AATTTGACTAACGCTAAGGCTGTATTTGCCCA-3′	
d: AgnCOMPRv	5′-GGATCCTCAAACATCCACGCTGGACCCAAC-3′	

1 Annealing temperature 53°C.

2 Annealing temperature for overlap extension reactions 55°C (AB, CD and AD).

Both plasmids were used to transform *S. pombe* 1252 (*agn1* mutant strain) as described in Suga and Hatakeyama [47], and transformants selected on EMM plates without leucine. As controls, pREP3X::*agn1* (expression vector containing the *S. pombe agn1* gene, positive control) and pREP3X (empty vector, negative control) were used. Transformants were evaluated by PCR using the primers listed in Table 1.

Complementation of *S. pombe* 1252 by the *P. brasiliensis AGN1* gene was followed by observation of calcofluor white stained cells in a fluorescence microscope Leica DM2000 equipped with H3 filter. Photographs of fluorescent images were taken with a Leica DFC310 FX digital camera, using an immersion objective with 100X magnification. For microscopic observation, 50 µl of cell suspension was mixed with 50 µl of 1 µg/µl calcofluor white (Sigma, F3543). The mixture was smeared onto slides plates pre-treated with 20 µl of 0.1% polylysine, air-dried, and washed with PBS. To quantify the degree of complementation, sedimentation assays were performed as in [15].

## Results

### *P. brasiliensis* AGN1 Sequence and in Silico Analysis

The *P. brasiliensis AGN1* gene has three exons that account for a putative coding region of 1495 bp, separated by two introns, all confirmed by comparison of the sequence of the RT-PCR product with the corresponding genomic sequence (Figure S1). It encodes a predicted protein of 456 amino acids (Figure S1), with high identity to fungal glucanases belonging to the glycoside hydrolase family 71 (GH-71) (*Neosartorya fischeri* 77%, *A. fumigatus* 76%, *A. niger* 76%, *A. nidulans* 74%).

In silico analysis of the deduced protein shows a signal peptide corresponding to the 21 initial amino acids, and a main domain homologous to the GH-71 family, which extends from residues 23 to 432 (Figure S1), similar to glucanases from *S. pombe* and *A. nidulans* [15], [16], [18]. It presents an estimated mass of 51.2 kDa, and an isoelectric point of 7.1. Also, putative sites for post-translational modifications are present. A hydropathic profile plot shows that the Agn1p sequence is predominantly hydrophilic except for three slightly hydrophobic areas, with no transmembrane domains (not shown).

**Figure 1:**
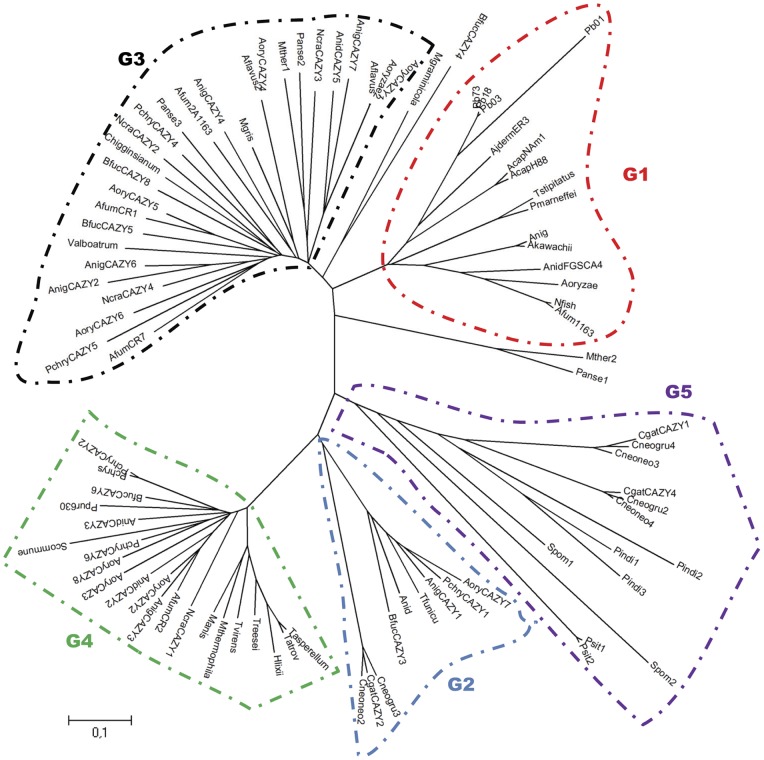
Phylogenetic tree of relatedness of family 71 of fungal glycosyl hydrolases (GH-71). The Mega 4 software package was employed, using ClustalW for sequence alignment. Construction of the phylogenetic tree was done by the neighbor-joining method using 1000 replications. Five groups (G1– G5) are distinguished. *P brasiliensis* Agn1p (labeled Pb73) is located in group G1. The groups are: G1 (red), G2 (blue), G3 (black), G4 (green), and G5 (purple). GenBank accession numbers of sequences, and names of fungal species used for construction of the tree are displayed in Table 2.

A search in the *P. brasiliensis* genome database (http://www.broadinstitute.org/) shows that *AGN1* is the only gene in the *P. brasiliensis* genome related to the hydrolysis of α-1,3-glucan. A Clustal analysis was performed that included 90 complete amino acid sequences of fungal glucanases present in GenBank and CAZy databases (http://www.cazy.org/GH71_eukaryota.html), grouping *P. brasiliensis* Agn1p into the glycoside hydrolase family 71 (Table 2, Figure 1). Variations among amino acid sequences allow us to propose a subdivision in the family 71 of glycoside hydrolases into 5 sub-groups (G1, G2, G3, G4 and G5; Figure 1).

**Table 2:** Sequences used for alignments and phylogenetic tree construction.

Abbreviation	Organism	GenBank ID
Mgris	*Maganaporthe oryzae*	XP001410317.1
Aoryzae	*Aspergillus oryzae* strain RIB40	XP_003190188.1
Aoryzae2	*Aspergillus oryzae* strain RIB40	XP001817591.2
AjdermER3	*Ajellomyces dermatitidis*	EEQ89186.1
AfumCR7	*Aspergillus fumigatus*	XP748780.1
Akawachii	*Aspergillus kawachii* strain IFO4308	GAA88202.1
Tstipitatus	*Talaromyces stipitatus* strain ATCC10500	EED20417
AfumCR1	*Aspergillus fumigatus*	XP001481700.1
Afum1163	*Aspergillus fumigatus*	EDP51240.1
Pb73	*Paracoccidioides brasiliensis*strain Pb73	EF679780*
Pb18	*Paracoccidioides brasiliensis*strain Pb18	PADG-07461.1
Pb03	*Paracoccidioides brasiliensis*strain Pb03	PABG-04011
Pb01	*Paracoccidioides brasiliensis* strain Pb01	PAAG-04206
AcapNAm1	*Ajellomyces capsulatus* strain NAm1	XP001541955.1
AcapH88	*Ajellomyces capsulatus* strain H88	EGC44045.1
Nfish	*Neosartorya fischeri*	XP001266141.1
Anig	*Aspergillus niger*	XP001393938.2
Pchrys	*Penicillium chrysogenum* Wisconsin 54-1255	XP002558559.1
Ppur630	*Penicillium purpurogenum*	AAF27912.1
Pmarneffei	*Penicillium marneffei* strain ATCC 18224	EEAAA2220869
AfumCR2	*Aspergillus fumigatus*	XP_749530.1
Afum2A1163	*Aspergillus fumigatus* strain A1163	EDP47950.1
Hlixii	*Hypocrea lixii*	CAC80493.1
Tasperellum	*Trichoderma asperellum*	CAH04880.1
Tatrov	*Trichoderma atroviride* strain IMI20640	EHK46766.1
Treesei	*Trichoderma reesei* strain QM6a	EGR50065
Tvirens	*Trichoderma virens* ATCC 42464	EHK24030.1
AnidFGSCA4	*Aspergillus nidulans* strain FGSCA4	CBF74212.1
Anid.	*Aspergillus nidulans* FGSCA4	CBF84404
Spom1	*Schizosaccharomyces pombe*	NP001018296.1
Spom2	*Schizosaccharomyces pombe*	XP001713124.1
Aflavus	*Aspergillus flavus* strain NRRL3357	XP_002372708.1
Aflavus2	*Aspergillus flavus* strain NRRL3357	XP_002376817.1
Mgraminicola	*Mycospherella graminicola* strain IP0323	EGP82959.1
Mthermophila	*Myceliophthora thermophila*	XP_003666470.1
Manis	*Matarhizium anisopliae* strain ARSEF23	EFY96730.1
Chigginsianum	*Colletotrichum higginsianum*	CCF39274.1
Valboatrum	*Verticillium albo-atrum*	XP_003004949.1
AnidCAZY2	*Aspergillus nidulans*	EAA59998
AnidCAZY3	*Aspergillus nidulans*	EAA64374
AnidCAZY5	*Aspergillus nidulans*	EAA64724
AnigCAZY1	*Aspergillus niger*	CAK44966
AnigCAZY2	*Aspergillus niger*	CAK44988
AnigCAZY3	*Aspergillus niger*	CAK39658
AnigCAZY4	*Aspergillus niger*	CAK96739
AnigCAZY6	*Aspergillus niger*	CAK48462
AnigCAZY7	*Aspergillus niger*	CAK42453
AoryCAZY1	*Aspergillus oryzae*	BAE55589
AoryCAZY2	*Aspergillus oryzae*	BAE56438
AoryCAZ3	*Aspergillus oryzae*	BAE57518
AoryCAZY4	*Aspergillus oryzae*	BAE59070
AoryCAZY5	*Aspergillus oryzae*	BAE62804
AoryCAZY6	*Aspergillus oryzae*	BAE63147
AoryCAZY7	*Aspergillus oryzae*	BAE63239
AoryCAZY8	*Aspergillus oryzae*	BAE64542
BfucCAZY3	*Botryotinia fuckeliana*	CCD46319
BfucCAZY4	*Botryotinia fuckeliana*	CCD54886
BfucCAZY5	*Botryotinia fuckeliana*	CCD47426
BfucCAZY6	*Botryotinia fuckeliana*	CCD48323
BfucCAZY8	*Botryotinia fuckeliana*	CCD51144
CgatCAZY1	*Cryptococcus gattii*	ADV21435
CgatCAZY2	*Cryptococcus gattii*	ADV25747
CgatCAZY4	*Cryptococcus gattii*	ADV23630
Cneogru2	*Cryptococcus neoformans var. grubii*	AFR96695
Cneogru3	*Cryptococcus neoformans var. grubii*	AFR98649
Cneogru4	*Cryptococcus neoformans var. grubii*	AFR93842
Cneoneo2	*Cryptococcus neoformans var. neoformans*	AAW47079
Cneoneo3	*Cryptococcus neoformans var. neoformans*	AAW42417
Cneoneo4	*Cryptococcus neoformans var. neoformans*	AAW44487
Mther1	*Myceliophthora thermophila*	AEO54485
Mther2	*Myceliophthora thermophila*	AEO59198
NcraCAZY1	*Neurospora crassa*	EAA29582
NcraCAZY2	*Neurospora crassa*	CAB92025
NcraCAZY3	*Neurospora crassa*	EAA29099
NcraCAZY4	*Neurospora crassa*	EAA30893
PchryCAZY1	*Penicillium chrysogenum*	CAP80377
PchryCAZY2	*Penicillium chrysogenum*	CAP80960
PchryCAZY4	*Penicillium chrysogenum*	CAP92350
PchryCAZY5	*Penicillium chrysogenum*	CAP83026
PchryCAZY6	*Penicillium chrysogenum*	CAP94862
Psit1	*Picea sitchensis*	ACN40311
Psit2	*Picea sitchensis*	ACN40737
Pindi1	*Piriformospora indica*	CCA70335
Pindi2	*Piriformospora indica*	CCA71563
Pindi3	*Piriformospora indica*	CCA70334
Panse1	*Podospora anserina*	CAP61754
Panse2	*Podospora anserina*	CAP67746
Panse3	*Podospora anserina*	CAP71798
Tfunicu	*Tallaromyces funiculosus*	CAD48301
Scommune	*Schizophyllum commune*	XP_003026950

* Obtained in this work.

∼From *Paracoccidioides brasiliensis* sequence data bank: http://www.broadinstitute.org/annotation/genome/paracoccidioides_brasiliensis/MultiHome.html.

### AGN1 Transcription Analysis under Horse Serum Supplementation

**Figure 2:**
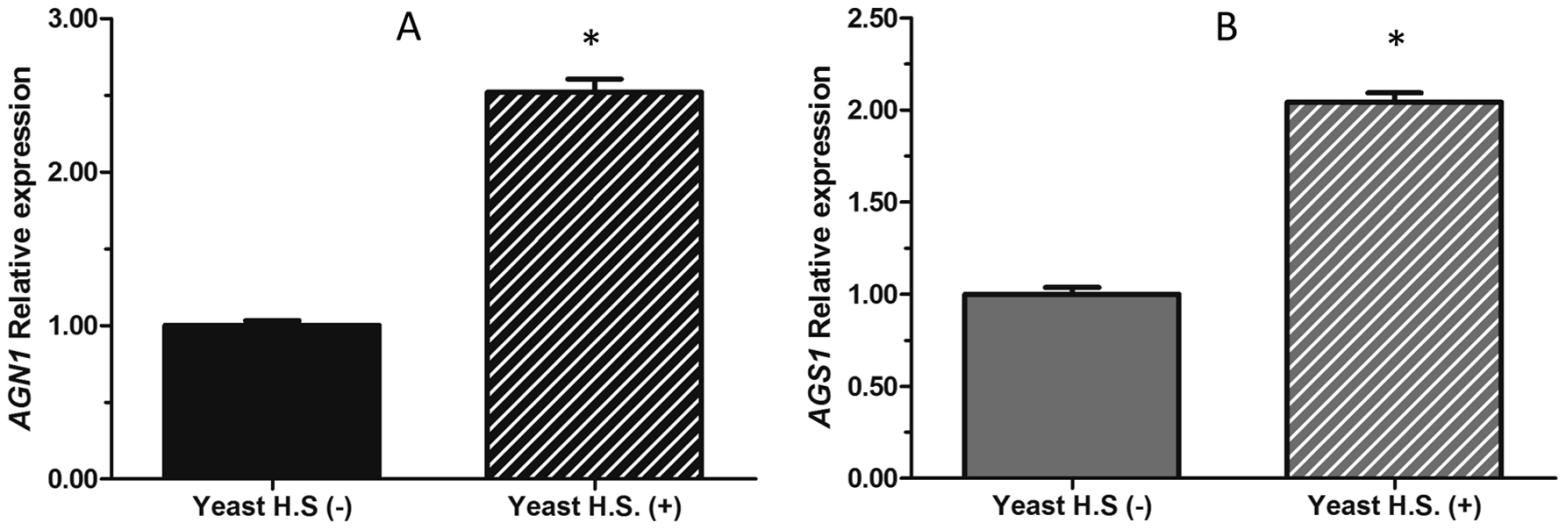
Expression analysis of *P. brasiliensis AGN1* and *AGS1*, under horse serum supplementation. Transcriptional levels were measured by qRT-PCR. Growing *P. brasiliensis* yeast phase supplemented with horse serum (HS), induces a statistically significant increase in the relative expression of *AGN1* (A) *and AGS1* (B), when compared to a control grown without HS. Yeast H.S. (-) (cultured without horse serum), Yeast H.S. (+) (cultured with horse serum). Error bars represent the standard deviation. (*) Turkey-Kramer test between Yeast H.S.(-) and Yeast H.S.(+); P-value <0.05. Experiments were done by triplicate.

Supplementation of growth medium with 5% horse serum (HS) has been reported as a booster for α-1,3-glucan synthesis in *P. brasiliensis* [12]. A qPCR expression analysis of *P. brasiliensis AGN1* and *AGS1* (encoding for α-1,3-glucan synthase, [12]) showed that their transcriptional levels were sharply increased in the presence of HS (Figure 2A and 2B), which agrees with the reported increase in cell wall α-1,3-glucan under supplementation of the culture medium with HS [12].

### Agn1p Heterologous Expression, and Biochemical Characterization

**Figure 3:**
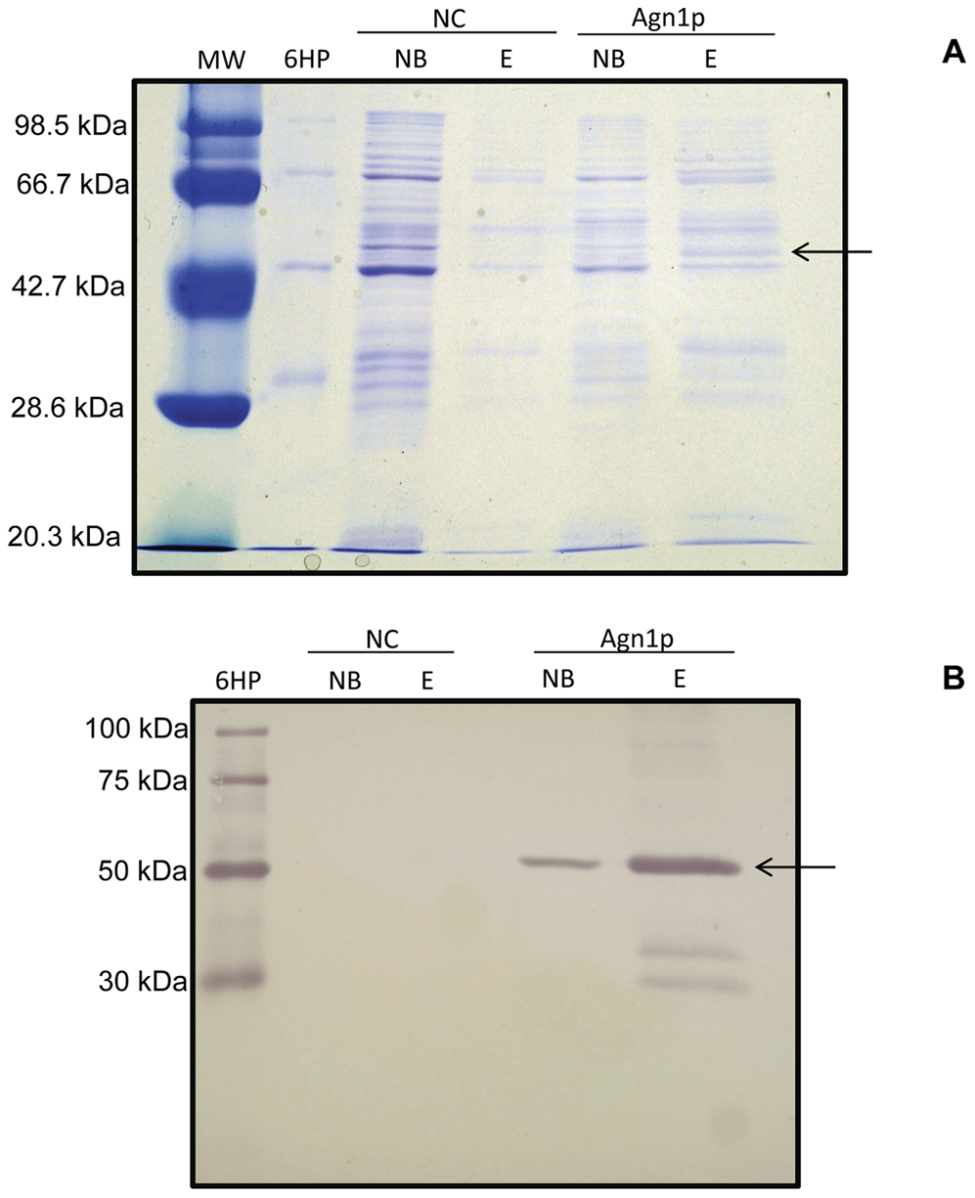
SDS-PAGE, and Western analysis of *P.* *brasiliensis* Agn1p. Ni-NTA-purified Agn1p from cell lysates of *E. coli* transformed with of pQE-30Xa::*AGN1* (Agn1p), and with the empty pQE-30Xa expression vector as negative control (NC) were separated by SDS-PAGE and stained with coomasie blue (A). The Ni-NTA-purified lysates were blotted on a nitrocellulose membrane and the His-tagged *P. brasiliensis* Agn1p (Agn1p) visualized using an anti RGS-His antibody (B). E stands for eluate, and NB for unbound material. MW: molecular weight marker. 6HP: 6xHis Ladder. Black arrow signals Agn1p position in both panels.

Protein expression of *P. brasiliensis α*-glucanase (Agn1p) was performed, using *E. coli* as the expression host. The cDNA without the signal peptide coding sequence was cloned into the pQE30Xa plasmid in frame with the His-tag present in the commercial plasmid, to produce the pHV2 vector. Induction of protein expression was obtained by addition of IPTG and the protein purified by affinity chromatography. A SDS-PAGE of the purified Agn1p-His showed a single band with an estimated molecular mass of 51.8 kDa (R^2^ = ∼0.98; Figure 3A and 3B), in close agreement with its calculated molecular mass of 51.2 kDa. A western analysis using antibody directed to the RGS-His epitope, confirmed that the band corresponds to the purified protein fused to the histidine tag (Figure 3B). A lower molecular weight band can also be seen, which may correspond to the degradation of Agn1p at the C-terminus, because the recorded signal shows the presence of RGS-His epitope located at the N-terminal region.

As substrate for enzymatic activity assays of Agn1p, soluble carboxymethylated α-1,3-glucan (SCMG), chemically modified from purified *P. brasiliensis* cell wall α-1,3-glucan, was used [48]. The carboxymethylation reaction was confirmed by infrared spectroscopy (IR) and ^13^C-NMR analysis (Figure S2). IR patterns showed a characteristic carbonyl signal (1780–10 cm^−1^) and the presence of glucose residues linked by α-1,3 bonds (∼918 and 840 cm^−1^) (data not shown). ^13^C-NMR clearly showed the corresponding signal of carbonyl groups (178.11 ppm; [39]), α-1,3- linkages were confirmed by peaks at 99.30, 80.02, 71.52, 70.03, 69.82, and 60.25 ppm (Figure S2) as reported in [49].

**Figure 4:**
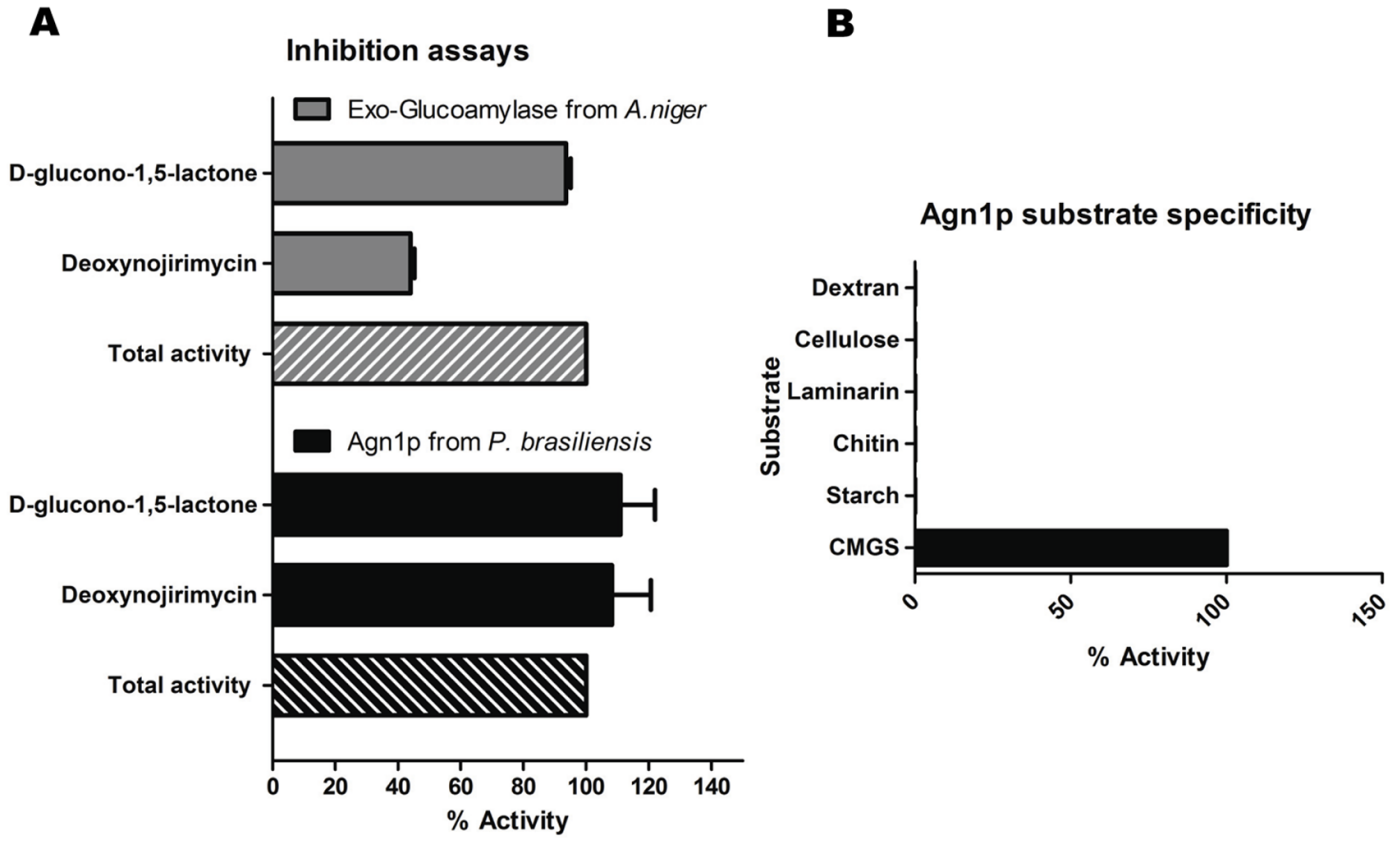
*P.* *brasiliensis* Agn1p is a specific endo α-1,3-glucanase. (**A**) Inhibition profile of exo-glucoamylase from *A. niger* (gray) and endo-α-1,3-glucanase from *P. brasiliensis* (black). Note that none of the indicated inhibitors reduced Agn1p-his activity significantly, even at a high concentration of 250 µM. (**B**) Agn1p substrate specificity. Purified Agn1p-his was incubated with the indicated substrates at 1 mg/ml. Reactions were carried out at optimal conditions by triplicate.

**Figure 5:**
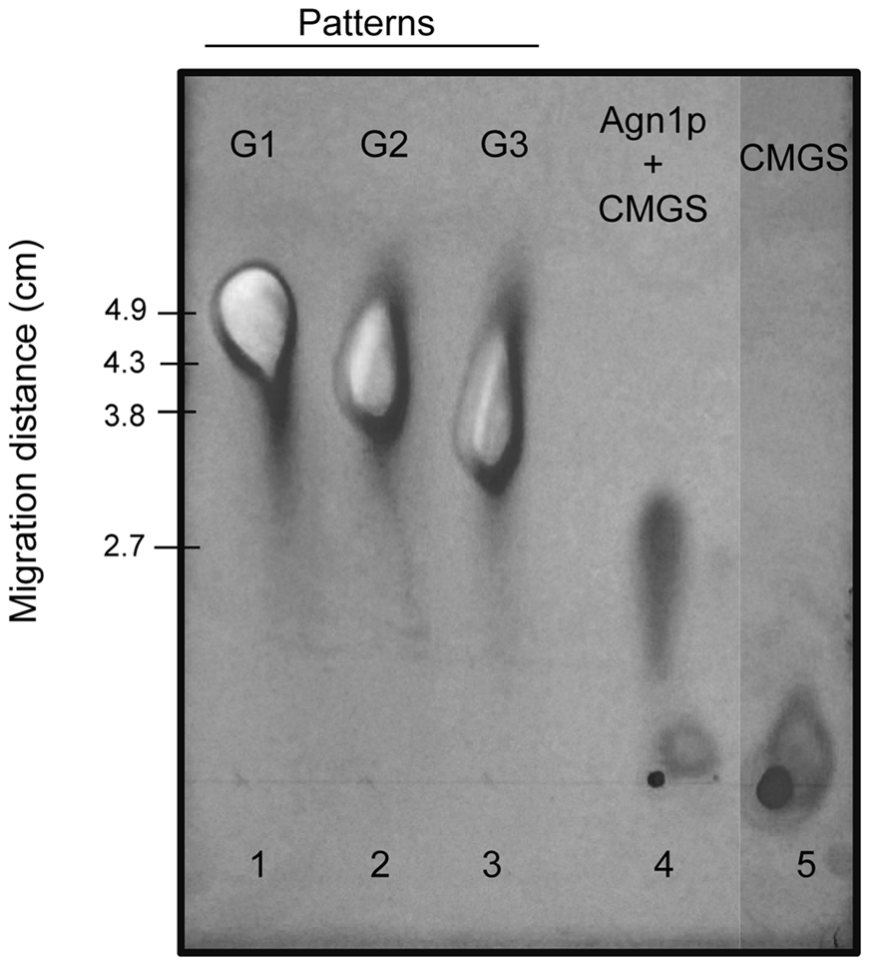
Thin Layer Chromatography (TLC). *P. brasiliensis* Agn1p was incubated for 1 h with CMGS. Lanes: 1, glucose (G1); 2, maltose (G2); 3, maltotriose (G3); 4, Agn1p incubated with GMGS; 5, CMGS.

Agn1p was only active against *α*-(1,3)-glucan (SCMG) when tested against a battery of glucose or glucosamine polymers (laminarin, starch, cellulose, chitin and dextran) (Figure 4B). Optimal reaction conditions for *P. brasiliensis* Agn1p were established at 1 h as pH 5.0 and 40°C (not shown). No inhibitory effect was observed upon Agn1p-his pre-incubation with inhibitors of exo-catalytic hydrolases (1-deoxynojirimycin and D-glucono-1,5-lactone) (Figure 4A). Endo-catalytic activity of AGN1 was determined by TLC analysis (Figure 5), where heptasaccharides (R^2^ = ∼0.9786) were the main hydrolysis products.

### AGN1 from *P. brasiliensis* Complements the Septation Phenotype of S. *pombe* agn1Δ Mutant

**Figure 6:**
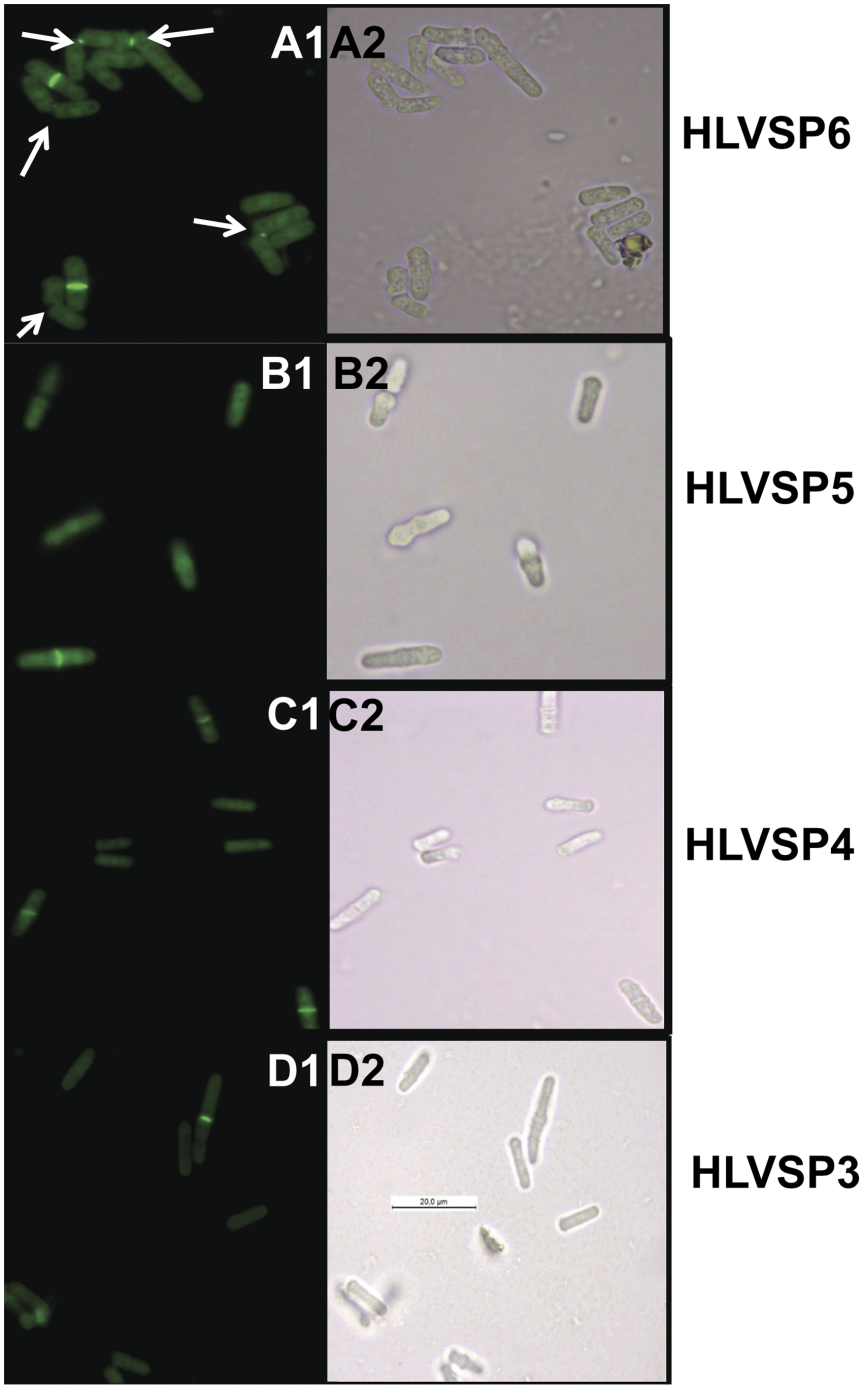
Complementation of *S. pombe agn1*Δ with the *P. brasiliensis AGN1* gene. *S. pombe ags1*Δ was complemented with pHV3, which contains the complete *P. brasiliensis AGN1* gene, including its original signal peptide coding region (*S. pombe* strain HLVSP3) (D1, D2) or pHV4, which includes a chimeric *P. brasiliensis AGN1*, whose signal peptide coding region was substituted by the *S. pombe agn1* signal peptide coding region (*S. pombe* strain HLVSP4) (C1, C3). In both cases, the plasmids restored the wild type phenotype. As positive control, plasmid pREP3X-*agn1^+^*, which includes the complete ORF from the *S. pombe agn1^+^* gene, was transformed into *S. pombe ags1*Δ (HLVSP5) (B1 and B2). Negative control consisted of *S. pombe ags1*Δ transformed with the empty vector pREP3X (HLVSP6) (A1, A2). White arrows point to the defect in separation at the tip of the daughter cells. Left panel show cells stained with calcofluor white (A1, B1, C1, and D1). Bar 20 µm.

For complementation, two different plasmids were introduced into *S. pombe agn1* null mutant strain 1252: (a) pHV3, containing the complete *P. brasiliensis AGN1* gene, including its original signal peptide coding region (*S. pombe* strain HLVSP3), and (b) pHV4, which includes a chimeric *P. brasiliensis AGN1*, whose signal peptide coding region was substituted by the *S. pombe agn1* signal peptide coding region, constructed by PCR overlap extension (*S. pombe* strain HLVSP4). As positive control, *S. pombe* HLVSP5, containing plasmid pREP3X-*agn1^+^*, which includes the complete ORF from the *S. pombe agn1^+^* gene, was used. Negative control consisted of *S. pombe* 1252 transformed with the empty vector pREP3X, (HLVSP6 strain). Cells were analyzed by calcofluor white staining, confirming that the strains carrying the *agn1^+^* and *AGN1* ORFs were able to suppress the separation defect shown by the *S. pombe* 1252 mutant (Figure 6), a result confirmed by sedimentation assays (Table 3).

**Table 3:** Sedimentation analysis of *S. pombe* strain 1252 (*agn1*Δ), complemented with *AGN1* from *P. brasiliensis*.

		Sedimentation time (min.)	
Genotype	Strain	80% of initial OD_595_	50% of initial OD_595_
*agn1*Δ [pREP3X]	HLVSP6	11.66±1.44	20±2.5
*agn1*Δ [pREP3X-agn1^+^]	HLVSP5	>30	>30
*agn1*Δ [pHV4]	HLVSP4	26±1	>30
*agn1*Δ [pHV3]	HLVSP3	25.17±2.25	>30

The values presented are the mean ± SD of four individual experiments.

## Discussion

*P. brasiliensis* strain Pb-73 has a single α-1,3-glucanase-encoding gene (*AGN1*) interrupted by two introns (accession number EF679780), whose product, Agn1p, is 77% identical to other fungal α-1,3-glucanases. *P. brasiliensis* Agn1p has a molecular mass of ∼51 kDa after SDS/PAGE; according to its amino acid sequence it can be classified into the poorly characterized family 71 of glycoside hydrolases (GH-71). A clustal alignment of *P. brasiliensis* Agn1p with other fungal GH-71 allows us to infer the location of 5 conserved residues, specifically aspartic and glutamic acids (**D** and **E** respectively), which may correspond to amino acids involved in the acid-base catalytic mechanism [50], [51]. Figure 1 shows a phylogram of relationships between different fungal GH-71. Five clearly differentiated clusters can be observed, which allow us to propose a subdivision of fungal GH-71 into at least five groups (G1 to G5) (Figure 1, Table 2), with *P. brasiliensis* Agn1p clustering into G1. G3, G4 and G5 share the conserved sequence (**T/S)WND**, while G4 and G2 shared the consensus sequence: **FALN**. It should be noted that glucanases that exhibit a mutan-binding domain (MBD) are grouped exclusively within the group G4 (*Hypocrea lixii*, *Trichoderma asperellum*, *Penicillium purpurogenum*), showing high identity to *T. harzianum* MBD (from 51 to 86%, [59]. Every group also presents specific conserved signatures: **SFDY, SWDAWP, WFYT, KNWLW, GTTGN** for group G1; **ISFD, VSTF(V/I)GD, GESHYI, YMAPVS, KNWVF** for group G2; **T(F/I)EG** for group G3; **GIDAFALNIG, F(A/V)SF, SKNW, (I/V)YWYR, G(I/L)YNFN** for group G4, (**S/N)(L/F)D(M/V), F(A/V)LN** for group G5.

The first 20 amino acid of *P. brasiliensis* Agn1p are predicted to be a signal peptide, suggesting the location of Agn1p towards the *P. brasiliensis* membrane or cell wall, where the α-1,3-glucan, a virulence factor and the specific substrate for Agn1p, is located. This location is shared by most of the fungal α-1,3-glucanases so far studied [15], [17], [13], [16], [18]. In agreement with the presence of the signal peptide, computationally predicted post-translational modifications were found (Figure S1). Among them, a sequence for cellular adhesion, described in *P. brasiliensis, H. capsulatum, A. fumigatus, C. immitis,* for proteins that bind to the extracellular matrix [52], [53], [54], [55], [56], and an N-glycosylation site, reported to play a role in post-translational modification of *Candida albicans* cell wall proteins involved in cell adhesion processes. Despite those possible post-translational modifications, we were able to achieve the purification of functional Agn1p from heterologous expression in *E. coli,* showing that in the absence of post-translational modifications (due to intracellular heterologous expression) the glucanase activity remains, as was recently reported for a recombinant glucanase from *T. harzianum* expressed also in *E. coli* [57]. Such glucanase has a specific activity of 0.097 U/mg, while the *P. brasiliensis* α-1,3-glucanase, measured at optimal conditions with SCMG as the soluble substrate, had a specific activity of 0.075 U/mg. It should be noted that the conditions used for carboxymethylation have been described as adequate to ensure solubility without alteration of the linear structure of the polysaccharide [39]. IR and ^13^C-NMR (Figure S3) spectra of SCMG indicate that carbonyl groups were properly added to the otherwise unchanged polysaccharide, data that support the effectiveness of the reaction, and the maintenance of an α anomeric configuration in the resulting SCMG [49]. *P. brasiliensis* Agn1p enzyme showed high specificity for its proposed natural substrate, cell wall α-1,3-glucan (SCMG, Figure 4B). The enzyme had an endo-catalytic activity, as deduced from TLC results (Figure 5, oligosaccharides as reaction products) and the lack of inhibitory effects by exo-catalytic inhibitors of hydrolases (Figure 4A). This high specificity and cutting pattern is shared with *S. pombe*, *P. purpurogenum* and *T. harzianum* glucanases [17].

Gene expression analyses by real-time PCR for both *AGN1* and *AGS1* in the Y phase (Fig. 2), showed significant increases (2 to 2.5 times transcript levels) in the expression of both genes when growing the pathogenic Y phase in the presence of horse serum, which boosts the synthesis of cell wall α-1,3-glucan, as previously reported [12]. This result suggests that the increased expression of *AGN1* in *P. brasiliensis* is related to an increase in cell wall α-1,3-glucan in the Y phase of this fungus.

Functionality of the *P. brasiliensis AGN1* gene was demonstrated by complementation of *S. pombe* strain 1252, an *agn1* null mutant. This mutation produces cell clumps due to the inability of mother-daughter cells to split, once the glucanase required for the hydrolysis of the septal α-1,3-glucan is unavailable. In *S. pombe,* the septum is formed by a primary septum (mainly β-1,3-glucan), surrounded by a secondary septum (a mixed structure of α-1,3-glucan, 1,6-branched 1,3-β-glucan, 1,6-β-glucan and galactomannans), through which septum degradation and cell separation starts. Therefore, *agn1* mutants are incapable of splitting, as shown with calcofluor white staining (Figure 6, A1-A2) [58]. The separation of the two daughter cells in *S. pombe* is initiated through secondary septum degradation; hence, the absence of α-1,3-glucanase activity prevents the splitting of the primary septum. Expression of *P. brasiliensis AGN1* into *S. pombe agn1*Δ, either with its original signal peptide-coding region or as a chimera with the *P. brasiliensis* signal peptide-coding region substituted by *S. pombe agn1* signal peptide-coding region, restored the wild type phenotype (Figure 6, C1,C2 and D1, D2; Table 3), and demonstrated the functionality of *P. brasiliensis AGN1*. This fact, plus the high specificity shown by *P. brasiliensis* α-1,3-endoglucanase, suggest the involvement of this enzyme in the yeast phase cytokinesis.

The fact that *P. brasiliensis* genome presents a single *AGN1* gene seems to be in consonance with the presence of a single α-1,3-glucan synthase (*AGS1*) gene recently reported [12]. Ags1p is associated with the synthesis of cell wall α-1,3-glucan, a proposed virulence factor in *P. brasiliensis*, and found to contribute to pathogenesis in *H. capsulatum* by concealing immunostimulatory β-glucans from detection by host phagocytic cells [5], [7]. Unlike the metabolism of chitin, which depends on up to seven different chitin synthases [59, 60. 61], and several chitinases [62], [63], the apparent simplicity of the mechanisms of synthesis and hydrolysis of *P. brasiliensis* α-1,3-glucan (one synthase, one hydrolase), and the fact that this polysaccharide is absent from the natural fungal host, leads us to propose both, its mechanisms of synthesis (by blocking it) and degradation (by stimulating it) as potential targets for the development of specific drugs against *P. brasiliensis*, which might result in the depression of fungal virulence, and allow the action of the natural immune response of the infected organism against the fungus.

## Supporting Information

Figure S1 ***AGN1*** **genomic sequence (gDNA) from** ***P.*** ***brasiliensis*** **strain Pb-73.** Highlighted in yellow, the deduced amino acid sequence of *P. brasiliensis* α-1,3-glucanase Agn1p. Highlighted in italics and bold, the putative start codon and the methionine residue attached, respectively. In red letters, 21 amino acids belonging to a putative signal peptide. In green, *AGN1* intron sequences, (their processing sites are underlined). Post-translational putative modification sites are highlighted in colored boxes: blue: cell adhesion; purple, N-glycosylation.
(TIF)

Figure S2 **SCMG^ 13^C-NMR spectra.** The box indicates the location of the signals corresponding to the carbonyl group, while the arrows point to the signature band of the α-1,3 configuration of both SCMG and α-1,3-glucan.
(TIF)
