# Supplementary figures and images for: Biochemical Characterization of Paracoccidioides brasiliensis α-1,3-Glucanase Agn1p, and Its Functionality by Heterologous Expression in Schizosaccharomyces pombe
Source: PLoS One. 2013 Jun 25;8(6):e66853. doi: 10.1371/journal.pone.0066853 (PMC3692533; doi:10.1371/journal.pone.0066853)

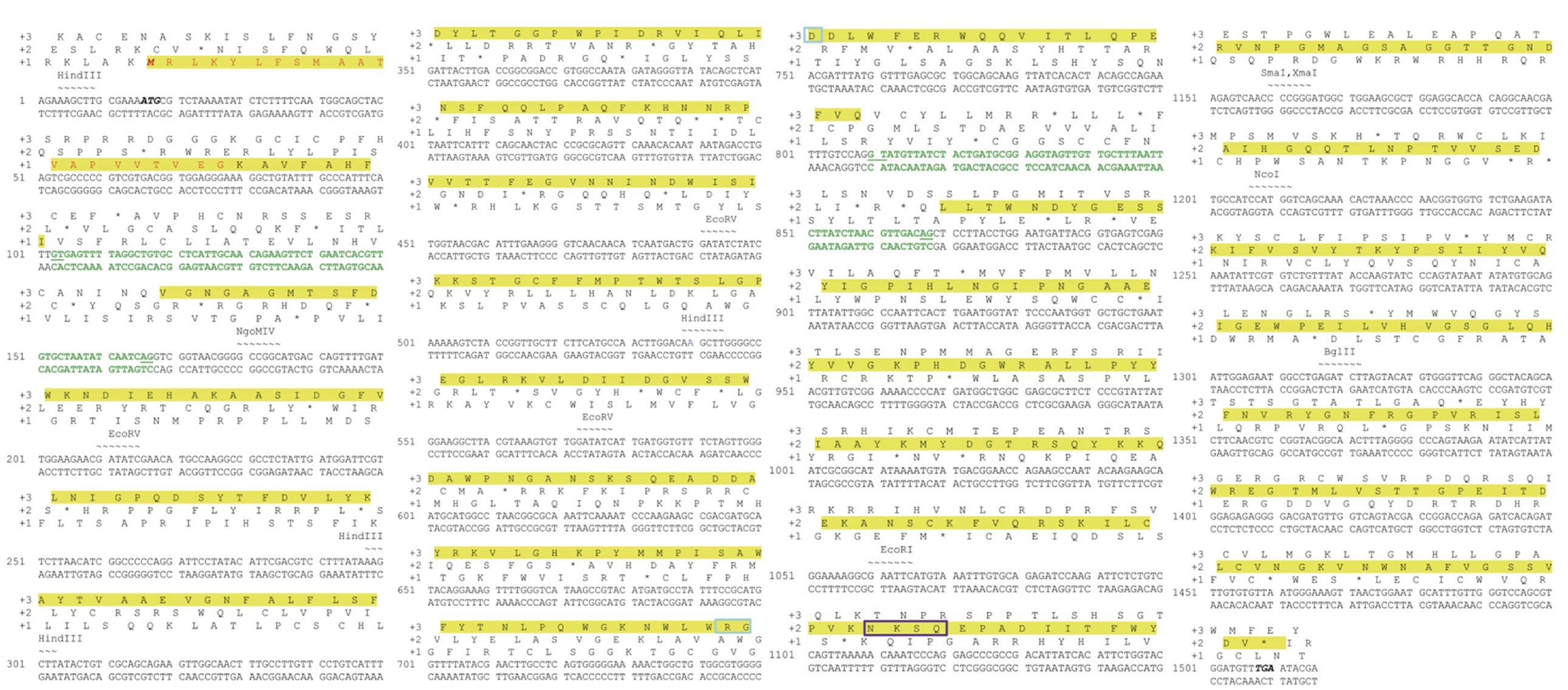

Supplement: Figure S1 — AGN1 genomic sequence (gDNA) from P. brasiliensis strain Pb-73. Highlighted in yellow, the deduced amino acid sequence of P. brasiliensis α-1,3-glucanase Agn1p. Highlighted in italics and bold, the putative start codon and the methionine residue attached, respectively. In red letters, 21 amino acids belonging to a putative signal peptide. In green, AGN1 intron sequences, (their processing sites are underlined). Post-translational putative modification sites are highlighted in colored boxes: blue: cell adhesion; purple, N-glycosylation. (TIF) [file pone.0066853.s001.tif]

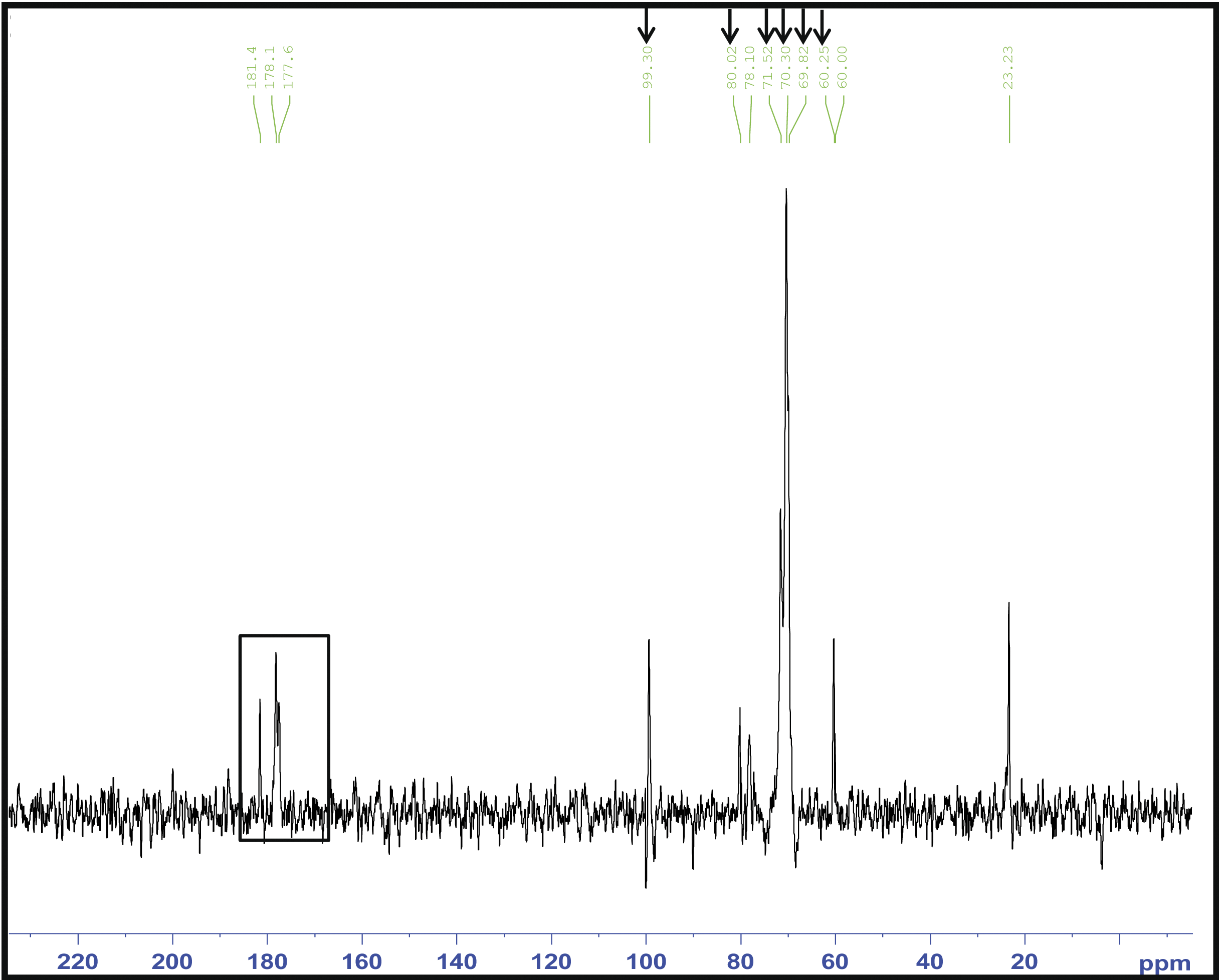

Supplement: Figure S2 — SCMG 13C-NMR spectra. The box indicates the location of the signals corresponding to the carbonyl group, while the arrows point to the signature band of the α-1,3 configuration of both SCMG and α-1,3-glucan. (TIF) [file pone.0066853.s002.tif]
